# The Role of Lectin Receptors and Their Ligands in Controlling Allergic Inflammation

**DOI:** 10.3389/fimmu.2021.635411

**Published:** 2021-04-30

**Authors:** Karin Peters, Marcus Peters

**Affiliations:** Department of Molecular Immunology, Ruhr-University Bochum, Bochum, Germany

**Keywords:** carbohydrate, immunomodulation, allergic inflammation, asthma, C-type lectic receptor

## Abstract

More than fifty c-type lectin receptors (CLR) are known and have been identified so far. Moreover, we know the group of galectins and sialic acid-binding immunoglobulin-type lectins that also belong to the carbohydrate-binding receptors of the immune system. Thus, the lectin receptors form the largest receptor family among the pathogen recognition receptors. Similar to the toll-like receptors (TLRs), the CLR do not only recognize foreign but also endogenous molecules. In contrast to TLRs, which have a predominantly activating effect on the immune system, lectin receptors also mediate inhibitory signals. They play an important role in innate and adaptive immunity for the induction, regulation and shaping of the immune response. The hygiene hypothesis links enhanced infection to protection from allergic disease. Yet, the microbial substances that are responsible for mediating this allergy-protective activity still have to be identified. Microbes contain both ligands binding to TLRs and carbohydrates that are recognized by CLR and other lectin receptors. In the current literature, the CLR are often recognized as the ‘bad guys’ in allergic inflammation, because some glycoepitopes of allergens have been shown to bind to CLR, facilitating their uptake and presentation. On the other hand, there are many reports revealing that sugar moieties are involved in immune regulation. In this review, we will summarize what is known about the role of carbohydrate interaction with c-type lectins and other sugar-recognizing receptors in anti-inflammation, with a special focus on the regulation of the allergic immune response.

## Introduction

There are more than 180,000 entries in Pubmed containing the search terms carbohydrate and immunomodulation. Moreover, there are many patents proposing carbohydrates as immunomodulatory substances. However, some polysaccharides known to bind to CLR, such as β-glucans and arabinogalactans, are already on the market as consumer healthcare products. However, the mode of action of these carbohydrates is predominantly unclear. It is likely that many of these effects can be explained by the interaction of sugar motifs with lectin receptors of the immune system. One function of lectins is to facilitate the uptake of antigens, even though these molecules are more than simple uptake receptors. Several of these lectin receptors mediate signals since they possess distinct signaling motifs. In particular, CLR expressed on myeloid cells play a central role in innate immunity. The CLR are distinguished between those that have an ‘immunoreceptor tyrosine-based activation motif’ (ITAM) and those with an ‘immunoreceptor tyrosine-based inhibition motif’ (ITIM). Thus, they can mediate activating or inhibiting signals to the immune system depending on the ligand. In addition to CLR, we also know the group of galectins and the group of Siglecs (sialic acid-binding immunoglobulin-type lectins). Most receptors of the Siglec family also transduce signals *via* ITAM/ITIM domains, whereas galectins are somewhat different in that they are secreted proteins which bind to carbohydrates on the surface of other cells.

In this review, we will focus on the modulation of the allergic immune response of the airways by carbohydrate/lectin receptor interaction. There are several studies that focused on the role of lectin receptors and discussed carbohydrate structures on allergens as crucial factors in allergic sensitization (1, 2). On the other hand, there are known cases where signaling *via* c-type lectins is exploited by pathogens to suppress the immune response (3). Thus, knowing these interactions could pave the way for therapeutics that could be used for the suppression of allergic inflammation.

One initial event during allergic sensitization *via* the airways is that epithelial cells become activated and start to release cytokines, which attract and activate dendritic cells (4). After taking up and processing allergens, they migrate to the draining lymph nodes to activate T lymphocytes. Moreover, B cells produce allergen-specific immunoglobulin E (IgE), which binds to the surface of mast cells. During the effector phase, mast cells become activated to release histamine and pro-inflammatory mediators, eosinophilic granulocytes degranulate their toxic content and other innate immune cells contribute to inflammation. This type of allergic airway inflammation is the most common one that depends on the activity of T helper cells from the Th2 type (5). However, other endotypes have been described in the last few years that depend on Th17 cells, which attract neutrophils and promote severe disease courses (6). Many steps of this process could be modulated by carbohydrate/lectin interaction. Dendritic cells (DCs), mast cells, granulocytes and epithelial cells, for example, are known to express several different lectin receptors and could, therefore, be modulated by the appropriate ligands (**Table 1**). Thus, several of the important players of the allergic immune response could be modified by carbohydrates. There are many papers describing a potential benefit of treatment with different types of carbohydrates for protection against allergic sensitization and inflammation. The carbohydrates described range from synthetic oligosaccharides to support the growth of intestinal microflora (27) to polysaccharides isolated from plants (28), bacteria (29) or fungal (30) sources. However, the mode of action is clear only in a minority of examples. In this review, we will focus on these examples. We will discuss how stimulation of these receptors can modulate allergic inflammation and could hopefully be exploited in the future for allergy prevention.

**Table 1:** Carbohydrate recognition receptors (CRR).

CRR	Expression in immune cells	Ligands	General functions	Impact on allergic immune response	Ref.
Dectin-1	Neutrophils, monocytes, myeloid DCs, in humans also B cells and mast cells and eosinophils	β1,3glucan	Recognition of fungal and mycobacterial infections, pro-inflammation	The ligand curdlan lowers TH2 allergic immune response	(7, 8)
Dectin-2	Monocytes, dendritic cells, macrophages	High mannose, alpha-mannans	Recognition of fungal and mycobacterial infections, pro-inflammation	Induces cysteinyl leukotriene secretion, which attracts eosinophils and neutrophils, increases TH2 response	(9–11) (8)
DCIR	Monocytes, DCs, granulocytes, B cells, macrophages	Glycoepitopes on HIV, carbohydrates of endogenous proteins such as antibodies	Virus capture and transmission	IVIgs can reduce allergic airway disease *via* interaction of their carbohydrates with DCIR	(8, 12)
DC-SIGN	Monocytes, myeloid DCs	High mannose or fucose containing carbohydrates, e. g. Lipoarabinomannan of mycobacteria	Endocytotic activity, immunomodulatory role	Induces IL-10 producing dendritic cells, the ligand arabinogalactan reduces the activation of NFkb and T-cell stimulatory capacity	(8, 13)
Macrophage Mannose receptor	Macrophages, myeloid DCs, Langerhans cells	Mannose containing carbohydrates	Endocytotic receptor, recognition of microorganism, cross presentation on MHCI	unknown	(8)
DEC-205	Granulocytes, monocytes,T-cells, B-cells, dendritic cells, NK cells	n.d.	Endocytosis and antigen presentation on MHCII	Endocytotic receptor, when OVA is fused to the receptor by an antibody, the allergic response is supressed	(8, 14)
Siglec F (m)/Siglec 8 (h)	Eosinophils mast cells	sialosides that contain both sialic acid and sulphate, with the position of the sulphate being an important determinant of specificity	Mediate cell-cell interactions and signaling functions in the immune system	Suppresses eosinophilic inflammation and mast cell activation	(15–17)
Siglec E (m)/Siglec 9 (h)	Myeloid cells			Suppresses neutrophilic inflammation	(18, 19)
Galectin 1 (secreted)	Mainly dendritic cells and monocytes	Galactoside containing glycans	Play a role in inflammation, adaptive immune response, cell migration, autophagy and signaling	Protects from allergic asthma, limits eosinophil recruitment and promotes apoptosis of eosinophils	(20–22)
Galectin 3 (secreted)	Mainly Monocytes, myeloid DCs			Induces Tregs, dampens mucus production and subepithelial fibrosis in allergic asthma, lowers airway hyperresponsiveness to metacholine	(23–25)
Galectin 9 (secreted)	Monocytes, myeloid DCs, granulocytes			It can bind to glycoepitopes present on IgE, inhibiting the activation of mast cells by specific allergens	(26)

The expression of CRR in this table is given for immune cells only. Many of these receptors are also expressed on other cell types, as listed in www.proteinatlas.org. The general functions of these receptors are described for immune cells, moreover, examples for the modulation of the allergic immune response by these receptors is given in the table. h, human; m, mous; n.d., not determined.

## Dectin 1 – A Pattern Recognition Receptor Binding to β-Glucans

The CRD of Dectin-1 recognizes 1,3-β-glucans, which are frequently found in the cell walls of fungi, bacteria and some plants. Dectin-1 is expressed in both human and mice on DCs, monocytes, macrophages and neutrophil granulocytes, and exclusively in human also on B cells, mast cells and eosinophilic granulocytes (7, 31). In addition to its endocytotic activity, Dectin-1 has its own signaling cascade. It does not have a complete ITAM, which would contain two tyrosines. Nevertheless, after ligand-binding and phosphorylation of the tyrosine, the ‘spleen tyrosine kinase’ (SYK) is activated. Subsequently, the formation of the CARD9/Bcl10/Malt-1 complex leads to the activation of NF-κB and, thus, to the production of pro-inflammatory cytokines (32, 33). In the case of DCs, the activation of Dectin-1 leads to their maturation and, thus, to a strong T-cell stimulatory capacity. Dectin-1 activated-DCs initiate mainly a Th1 and Th17 immune response (34).

**Figure 1:**
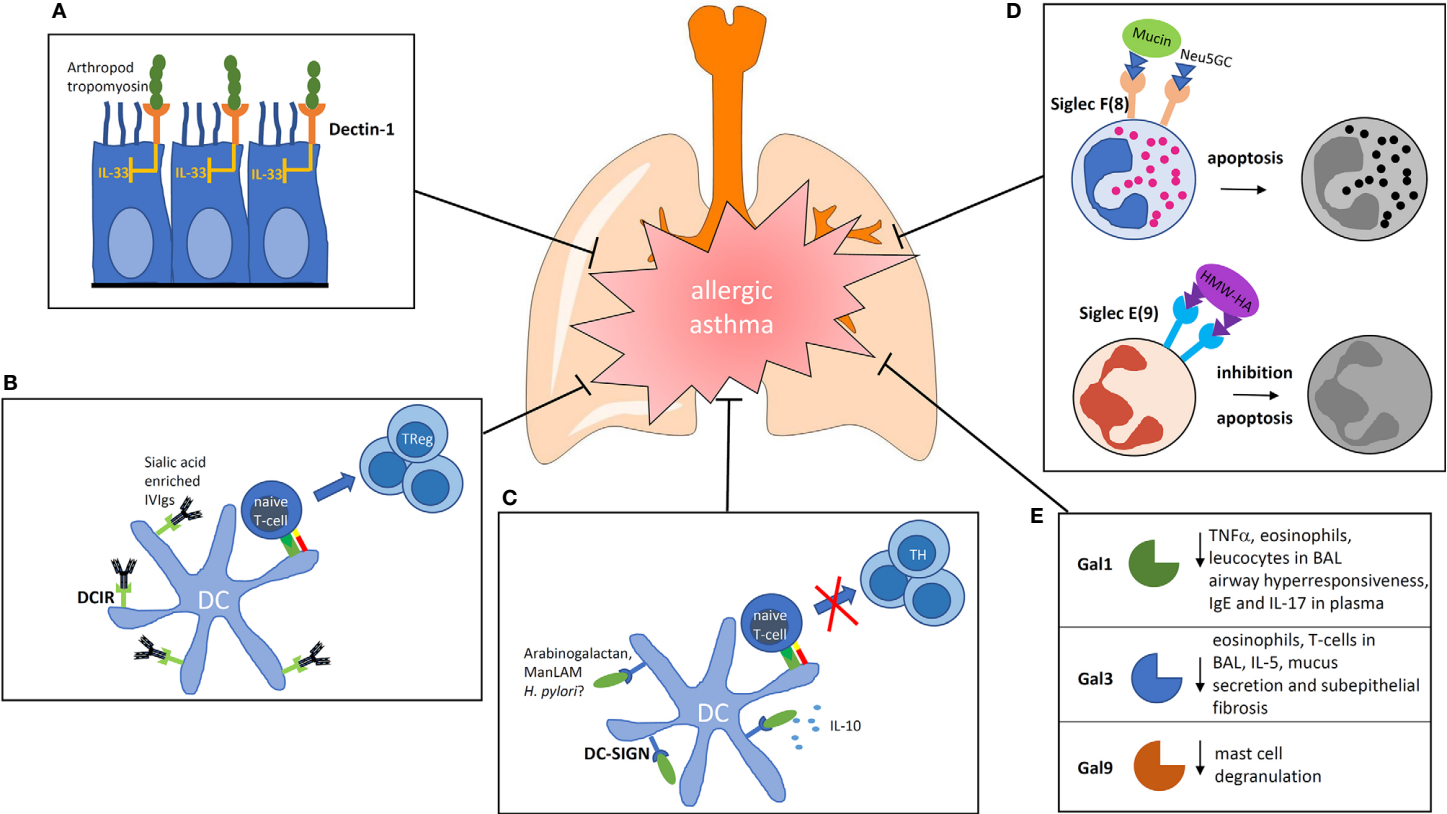
Impact of carbohydrate recognition receptors and their ligands on the modulation of allergic inflammation. **(A)** Binding of arthropod tropomyosin to Dectin-1 on airway epithelial cells inhibits IL-33 release. **(B)** DCIR is known for its inhibiting signaling properties. If sialic acid-rich antibodies are bound by DCIR on DCs they are able to induce regulatory T cells. **(C)** Ligation of DC-SIGN can induce prolonged IL-10 production. The ligand arabinogalactan reduces the activation of NFκb and T-cell stimulatory capacity of human DCs. **(D)** Siglecs are membrane spanning receptors with signaling properties. Siglec F signaling induces apoptosis of eosinophils, Siglec E inhibits neutrophils. **(E)** Galectins are secreted proteins which bind to the glycocalyx of cells. In the group of galectins, three members are known to lower the allergic response by different mechanisms. BAL, broncho alveolar lavage; Gal, Galectin; ManLAM, mannosylated Lipoarabinomannan; IVIg, intravenous Immunoglobulins; HMW-HA, high molecular weight hyaluronan; Neu5GC, N-Glycolylneuraminic acid.

Despite this immunostimulatory role of Dectin-1, there are some reports revealing a beneficial role of Dectin-1 stimulation in the prevention or control of allergic asthma. Invertebrate tropomyosin, for example, a ubiquitous arthropod-derived molecule, was shown to be a dectin-1 ligand that serves to restrain IL-33 release, thus, dampening type 2 immunity in healthy individuals (35). In this context, the immune modulation is likely to be mediated *via* an influence on the airway epithelium and not by a direct effect on DCs (**Figure 1A**). However, stimulation with a well-known Dectin ligand, namely Curdlan, also led to reduced allergy (36). In this paper, the authors showed that the activation of antigen-presenting cells is involved, which lead to the generation of the interleukin (IL)-10-producing T-helper *via* ICOS interaction.

By contrast, other reports showed that β-glucans may worsen allergic asthma by acting through Dectin-1 (37). In line with what is known about the polarizing activity of β-glucan-stimulated DCs, the authors observed that Curdlan induced a Th17 response with increased neutrophilic inflammation and exacerbation of the disease.

## Dectin 2 – A Pattern Recognition Receptor Binding to High Mannose Structures

Dectin-2 is described as representative for CLR, whose signaling pathway runs *via* a coupled ITAM that belongs to a neighbored Fc-receptor gamma chain (9). Dectin-2 is present on macrophages, monocytes and DCs (10). Its ligands are structures with a high mannose content and α-mannans, which are found in fungi (11). The activation of Dectin-2 leads to the recruitment and activation of SYK, which follows a similar signaling pathway as Dectin-1.

Dectin-2 is associated with allergic diseases mainly because house dust mite extracts are able to activate SYK *via* Dectin-2 and, thus, cause a rapid release of cysteinyl leukotrienes. This leads to a recruitment of eosinophilic and neutrophilic granulocytes and an amplification of the Th2-dependent allergic immune response (38).

In fact, there are no reports in the literature arguing for an anti-inflammatory role of Dectin-2 stimulation. Therefore it seems as if Dectin-2 stimulation is predominantly acting in a pro-inflammatory way and is, thus, triggering an allergic immune response.

## DCIR – Immune Inhibiting Receptors Binding to Endogenous Fucose or Mannose Containing Ligands

There is also a group of receptors which show inhibitory activity in addition to activating CLR. Dendritic cell immunoreceptors (DCIRs) have an ITIM instead of an ITAM domain. Two paralogs of this receptor exist in mice. The ‘dendritic cell immunoreceptor 1’ (DCIR1), which is expressed on monocytes, granulocytes, macrophages, DCs and B cells, and DCIR2, that seems to be more restricted to expression in cDCs, are both representative of this group. Fucose and mannose-containing glycans are ligands for DCIR (12).

Only one DCIR is described in human. No exogenous ligands other than HIV-1 have been described for human DCIR so far (39). The intracellular domain of DCIR is associated with the non-receptor tyrosine kinases SHP-1 and SHP-2 *via* ITIM (40).

DCIR2-deficient mice were shown to be more prone to autoimmunity and have a higher number of DCs underlining the immunoregulatory role of the receptor (41). It is known that intravenous injections of immunoglobulin (IVIg) of healthy donors leads to an alleviation of the disease in different inflammatory conditions in human (42). It was thought earlier that IVIg would act in the sense of passive immunization. However, it became increasingly clear that the glycosylation pattern of the Fc part of the antibodies injected plays an important role in immunomodulation (43). Interestingly, IVIg alleviates allergic airway disease through the interaction of its carbohydrates with DCIR in a mouse model (**Figure 1B**). The authors concluded from their study that IVIg interaction with DCIR induces a tolerogenic response (44). Thus, DCIR seems to be a valuable target for modulation of the allergic immune response.

## DC-SIGN – Pattern Recognition Receptor Binding to Fucose or Mannose Containing Antigens

Another well-known representative of the ITAM/ITIM independent CLR is DC-SIGN. This receptor has a CRD that recognizes mannose- and fucose-containing motifs. Accordingly, ligands for DC-SIGN are found in a variety of pathogens, such as Mycobacterium tuberculosis, HIV, measles virus, Helicobacter pylori, Candida albicans and Salp15 from tick saliva (45). In addition to its endocytotic activity, DC-SIGN has a distinct signaling cascade. After the binding of mannosylated lipoarabinomannan to DC-SIGN, Raf-1 becomes activated, which, in turn, initiates the phosphorylation and acetylation of NF-κB leading to a prolonged production of IL-10 (46). The IL-10-producing DCs are known for their tolerogenic phenotype, in accordance, it was shown that mannosylated lipoarabinomannan-stimulated DCs led to the generation of regulatory T-lymphocytes (47). However, different ligands for DC-SIGN seem to have different effects on DCs. Salp15, for instance, was shown to activate the ‘dual specificity mitogen-activated protein kinase,’ which, in turn, leads to the degradation of IL-6 and TNF-α mRNA (48). Different paralogs of the receptor exist in mice.

Although DC-SIGN seems to play an immunoregulatory role, there are only a few reports that studied the ability of DC-SIGN ligands to suppress unwanted immune reactions.

There are many reports from mouse models on the protective activity of Mycobacteria and their extracts on allergy protection. However, it is not clear whether murine homologues of DC-SIGN are involved in this protection. One homologue of human DC-SIGN in mice is SIGNR1. This receptor was shown to be involved in the induction of oral tolerance to mannosylated antigens in mice (49). Furthermore, it was shown that extracts of H. pylori protect against allergic asthma in a mouse model by the induction of IL-10-producing DCs (50). Therefore, it is interesting to speculate that the protective effect is mediated *via* a murine DC-SIGN homologue, but, to date, there is no supporting data to prove this hypothesis.

In addition to the ligands discussed above, it was shown that plant arabinogalactan binds to DC-SIGN on the surface of human DCs. The binding resulted in a reduced activation of the transcription factor NFκB after TLR-stimulation (**Figure 1C**). This led to DCs with a reduced T-cell stimulatory capacity (13). Interestingly, arabinogalactan is a molecule that is found abundantly in extracts of cowshed dust and can inhibit allergic sensitization in a mouse model of asthma, suggesting that it might be also involved in the “farming effect” (51).

## Macrophage Mannose Receptor (MR) and DEC205 – Pattern Recognition Receptors for Endocytosis

Both receptors are involved in endocytosis, however, they do not contain an intracellular signaling motif. In addition to macrophages, MR is also found on some DC subpopulations. DEC205 has a broad expression pattern and it expressed on immature DCs, but it is now known to be expressed on endothelium and selected macrophage subpopulations (52). Endocytosis *via* MR leads to the cross-presentation of antigens *via* the ‘major histocompatibility complex’ I (53), whereas endocytosis *via* DEC205 leads to an enhanced presentation of the ingested antigen *via* major histocompatibility complex II (54). Regarding MR, it is not clear whether it is able to trigger an intracellular signaling cascade itself or this happens in cooperation with another CLR. However, it could be shown that the crosslinking of MR *via* antibodies (AK) led to cytokine production in DCs involving the anti-inflammatory cytokine IL-10 (55). No signaling pathways have been described for DEC205 so far.

After the discovery of DEC205 as an antigen delivery receptor, it was attempted to exploit DEC205 as a potential target to be used in vaccination approaches. Surprisingly, it was observed that antigens targeted into the endocytotic pathway *via* DEC205 induced a regulatory immune response that might inhibit the induction of immunity (56). Subsequently, this finding was exploited for the delivery of allergens by gene-based immunization using an adenoviral delivery of single chain antibodies directed against DEC205 fused to OVA to target the allergen towards DCs. This treatment resulted in the efficient suppression of an allergic immune response (14).

## Siglec – Glycoreceptors Binding to Sialylated Antigens

The Siglecs are transmembrane receptors that bind structures containing sialic acid. They are mainly expressed on immune cells and can be divided into three functional groups. The first group contains Siglec 1 and 4, without any known intracellular signal motifs. The second and largest group includes Siglec 2, 3 and 5 – 12 that contain an ITIM or ITIM-like motif intracellularly. Similar to CLR that contain an ITIM, the signaling cascade involves the recruitment of SHP-1 and SHP-2 and leads to an inhibition of other immunomodulatory signals, which are, for example, induced by the activation of TLRs (57). The third group of Siglecs consists of Siglec 14, 15 and 16 and is characterized by a positively charged residue in their transmembrane domain, through which they can associate with the negatively charged transmembrane domain of the DNAX-associated protein of 12 kDa (DAP12). The DAP12 carries an intracellular ITAM and can trigger a signaling cascade *via* SYK, similar to the CLR Dectin-1 or Dectin-2. Siglec H is transducing its signal in rodents *via* DAP12 (58).

Siglecs recognize both exo- and endogenous ligands. Their primary function is probably the regulation of immune responses. However, unlike CLR, this is not done *via* pathogen recognition since most pathogens are not sialinized.

The function of Siglec F (human functional paralog Siglec 8) has been explored *in vivo* using antibodies, knockout mice and models where the expression of specific ligands has been altered. Administration of agonistic anti Siglec-F antibodies, for example, reduced the number of eosinophilic granulocytes in blood by reducing the viability of the cells (15). Moreover, antibodies binding to Siglec F abrogate eosinophilic pulmonary inflammation and virtually eliminates lung remodeling in mouse models of chronic allergic asthma (16). Muc5b and Muc4 were identified as endogenous ligands for Siglec F because they carry sialylated glycan ligands (59). Purified mucin preparations carried sialylated and sulfated glycans that were able to induce apoptosis in mouse eosinophils. Muc5b-deficient mice displayed exaggerated eosinophilic inflammation in response to the intratracheal installation of IL-13.

Regarding Siglec E (human ortholog = Siglec 9), it was shown that Siglec E-deficient mice in an LPS-induced lung inflammation model exhibited exaggerated neutrophil recruitment (18). The authors concluded that signaling *via* Siglec E may control neutrophilic inflammation. Since severe neutrophilic asthma is an endotype that is difficult to treat, using agonistic antibodies or glycan ligands as new treatment options was discussed (19). One natural ligand that was identified is high molecular weight hyaluronan from the capsule polysaccharides of the pathogen group A of *Streptococcus.* Their binding to Siglec 9 suppresses the activation of neutrophilic granulocytes (60).

N-glycolylneuraminic acid (Neu5Gc) is a sialic acid that is expressed on nonhuman mammalian cells and glycoproteins and is not present in bacteria. It was proposed to be partially responsible for the “farming effect” because there is a relationship between the exposure toward Neu5Gc in a rural environment and protection against allergy. The immunomodulatory activity of Neu5Gc was confirmed by suppression of allergic airway inflammation in a murine model (61). An involvement of Siglec 8 as the binding receptor was discussed by the authors (**Figure 1D**) (62).

## Galectins – Soluble Glycoreceptors Recognizing Galactose-Containing Molecules

In addition to the group of CLR, there is a large number of other glycoreceptors that play a role in the immune response but are not considered PRRs. Galectins are small, soluble proteins that are expressed and secreted by different cells. Fifteen members of this protein family have been discovered so far (20). Functionally, they are able to influence the differentiation and survival of T cells and their interaction with DCs (63).

Gal-1-deficient mice exhibit an increased recruitment of eosinophils and T lymphocytes in the airways as well as elevated peripheral blood and bone marrow eosinophils relative to corresponding WT mice (21). Moreover, mice had an increased airway hyperresponsiveness and displayed significantly elevated levels of TNF-α in the lung tissue. The authors suggested from their results that Gal-1 can limit eosinophilic airway inflammation by inhibiting the migration and promoting apoptosis of eosinophilic granulocytes.

In order to evaluate Gal 1 as a potential therapeutic protein, Ly et al. have shown that the recombinant Gal 1 protects from allergic asthma in a mouse model (22). The immunomodulatory effects in the allergic lung were correlated with the activation of the extracellular signal-regulated kinase signaling pathway and downregulation of endogenous Gal-1. rGal-1 reduced the plasma concentrations of anti-OVA IgE and IL-17, therefore, it can be hypothesized that it may also act on the Th17 response involved in severe asthma.

Gal-3 gene-deficient mice also showed enhanced disease activity in a mouse model of asthma by Zuberi et al., arguing for a regulatory role in asthma (23). Intratracheal instillation of plasmid DNA encoding Gal-3 led to the normalization of the eosinophil and T-cell count in BALF and a strong inhibition of IL-5 mRNA in the lungs in a rat asthma model (24). It was shown in a chronic asthma model in mice that twelve weeks after the first intranasal allergen instillation, treatment with the Gal-3 gene led to an improvement in the eosinophil count and the normalization of hyperresponsiveness to methacholine. In addition, this treatment resulted in a reduced mucus secretion and subepithelial fibrosis (25), showing that Gal-3 also seems to have some therapeutic merit, although the mode of action in the asthma model was unclear. Regarding the mechanism, Tsai et al. showed that Gal-3 gene-deficient mice have more severe disease activity in a colitis model, indicating that Gal-3 may protect from inflammation (64). Moreover, the authors showed that mucosal inflammation was reduced in the colitis model by treating with Gal-3. There was strong evidence that regulatory T cells were induced by Gal-3.

Regarding Gal-9, it was shown that it can bind to glycoepitopes present on IgE, therefore, inhibiting the activation of mast cells by specific allergens (26). Moreover, the authors showed that Gal-9 attenuated asthmatic reaction in guinea pigs and suppressed passive-cutaneous anaphylaxis in mice, showing that Gal-9 may also be a potent modulator useful for the treatment of allergy (**Figure 1E**).

## Conclusion

We know several different lectin receptors playing a role in not only innate but also adaptive immunity. Some of them serve predominantly as PRR, others rather recognize the glycosylation pattern of self-molecules. Knowing these interactions may open the way to new therapeutics for immunostimulation in, for example, vaccination and for the regulation of exaggerated immune responses, such as allergic inflammation. The focus of this review was to sum up what is known about the carbohydrate/lectin interaction that could be exploited for immunomodulation to prevent or treat respiratory allergies.

We are still at the beginning of this exciting field of research that may contribute to the development of new allergy preventive drugs. However, there are many issues still to be addressed. Although we focused in this review on literature which showed allergy protection by carbohydrates, it cannot be generalized that treatment with carbohydrates always acts in a preventive manner. Many examples are known where carbohydrate/lectin receptor interaction was linked to allergic sensitization. Similar observations were described for experiments with TLR receptor ligands where contradictory results were also obtained, depending on the dose of ligand used and how the experiment was conducted. Therefore, it is very important to consider the quality of the carbohydrate ligand used, the experimental design and the ligand receptor interaction that is involved in all experiments performed.

Furthermore, the majority of the results summarized in this review come from experiments with mice. However, it is important to bear in mind that there are several differences between lectin receptors found in mice and their human homologues. The binding behavior and the signal transduction often differs between the receptors of the two species. Furthermore, several paralogous exist in mice which do not have comparable receptors in human. Thus, when immunomodulatory carbohydrates were identified in murine model systems, it is of particular importance to know the binding receptor to get an idea whether the substance would also act in human in a similar manner.

Due to the increased hygienic measures accompanying the recent virus pandemic, it can be expected that allergic diseases will continue to rise. Effective treatments to prevent allergic disease are urgently needed. There is still a long way until the first carbohydrates will enter into clinical trials, however, it is worth it. There are many examples of how the allergic immune response is modified by carbohydrates and many of them seem to have no pro-inflammatory properties. Therefore, this substance class would be ideally suited for prophylaxis.

## Author Contributions

All authors contributed to the article and approved the submitted version.

## Conflict of Interest

The authors declare that the research was conducted in the absence of any commercial or financial relationships that could be construed as a potential conflict of interest.
